# Comment on “Laboratory Measurement and Analysis of the Deteriorated Layer Permeability Coefficient of Soil–Cement Deteriorated in a Saline Environment”

**DOI:** 10.3390/ma13010196

**Published:** 2020-01-02

**Authors:** Rui Neves

**Affiliations:** Barreiro Technology School, Polytechnic Institute of Setúbal, R. Américo Silva Marinho, 2839-001 Lavradio, Barreiro, Portugal; rui.neves@estbarreiro.ips.pt

**Keywords:** permeation model, corrosion, water permeability, soil-cement

## Abstract

The derivation of a formula to compute the permeability coefficient in the commented paper assumes that the mass flow is homogeneous in a homogeneous layer of a specimen. This assumption is not correct when there is also, at least, one heterogeneous layer. Moreover, a mathematical lapse was found on one equation, that would prevent the right computation of the permeability coefficient, even if the assumption was correct. Although this does not invalidate the major conclusions of the study and has not an outstanding effect on the presented results, for the sake of rigor and sound background for future studies in this field, corrections to the published formulas and model are proposed.

## Comment

The investigation published in *Materials*, by Jin et al. [1], was found interesting and invited further analysis, that resulted in the present Comment. In fact, two issues were found.

The first issue consisted of a lapse in the development of the formula to compute the permeability coefficient, in a specimen with different media. The mistake occurred when the authors, after substituting Equation (6) in Equation (4), divided the resulting expression by *H*, to introduce the *R_H_* term. Besides the several ratios *d*/*H*, the ratio *H_m_*/*H* was amiss also taken as *R_H_*. Thus, Equation (7) of Reference [1] should be

(1) $$R_{a}k_{d}{}^{2}+\left(k_{0}-k_{c}-k_{0}R_{a}+2k_{c}R_{H}-2k_{c}R_{a}R_{H}\right)k_{d}-2k_{0}k_{c}R_{H}\left(1-R_{a}\right)=0.$$

Consequently, the formulas to compute parameters *A* and *B* of Equation (8) in Reference [1], are not correct (assuming that Equation (9) is intended to provide the formula for *A*). The correct formulas are

(2) $$A=\left(2k_{c}+\frac{k_{0}}{R_{H}}\right)R_{a}R_{H}+k_{c}-2k_{c}R_{H}-k_{0}$$

for Equation (9) of Reference [1], and

(3) $$\begin{array}{l} B={k_{0}}^{2}\left({R_{a}}^{2}{R_{H}}^{2}-2R_{a}R_{H}+1\right)+2k_{a}k_{c}\left[R_{a}\left(1-2R_{a}R_{H}\right)+2R_{H}-1\right]+\\ {k_{c}}^{2}\left[4R_{a}R_{H}\left(R_{a}R_{H}-2R_{H}+1\right)+4R_{H}\left(R_{H}-1\right)+1\right] \end{array}$$

for Equation (10) of Reference [1].

This mistake is made clear in the results for the soil–cement with a cement content of 10%, tested at 45 days. From Figure 10 of Reference [1], *k*_0_ and *k_c_* have the same value (near 0.32 × 10^−8^ cm/s), while *k*_d_ has a different value (near 0.17 × 10^−8^ cm/s, following Figure 12 of Reference [1]), that cannot be correct. If the permeability coefficients of the two different media of a specimen are different, then the permeability coefficient of the specimen shall lay between the permeability coefficients of the different media. If both media have the same permeability coefficient, then the permeability coefficient of the specimen will be equal to the permeability coefficient of the media. This will happen if Equations (2) and (3) are used (together with Equation (8) of Reference [1]). 

Secondly, a more important issue, that justifies this Comment, was judged to exist in the background of the derivation of the formula to compute the permeability coefficient in a specimen with two different media.

In the following, two layers of different media crossed by a perpendicular flow are designated as serial association (*SA*), and two layers of different media being crossed by mass in a flow that is parallel to both are designated as parallel association (*PA*).

**Figure 1:**
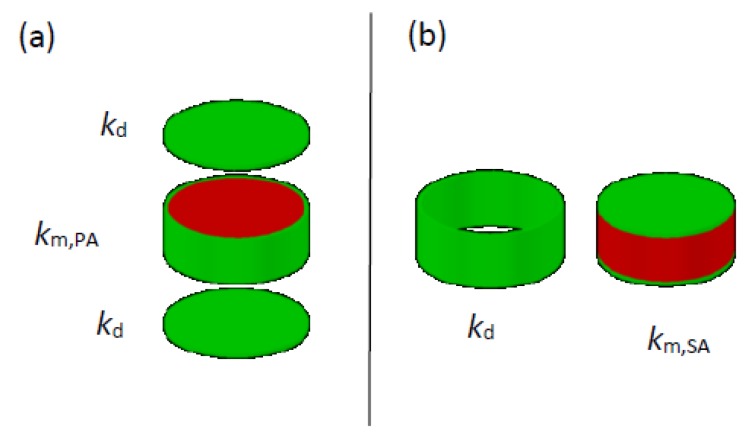
Approaches for permeation in heterogeneous media: (**a**) considered in Reference [1]; (**b**) suggested in this Comment.

Both (*SA* and *PA*) exist in a non-completely deteriorated soil–cement specimen. The question is in that Jin et al. [1] adopt an approach considering that the mass flow in a homogeneous layer is homogeneous as well, regardless of eventual heterogeneities in other layers previously crossed by mass. Actually, in a layer with *PA* there will be different flows, and the continuity of the flow(s) imposes that in subsequent layers, even though they are homogeneous, there will be different flows as well. Figure 1 depicts the approach adopted in Reference [1] and the approach suggested in this Comment.

Knowing that the overall permeability coefficient for permeation in *SA*, *k_m,SA_*, is

(4) $$k_{m,SA}=\frac{\sum L_{i}}{\sum \frac{L_{i}}{k_{i}}}$$

where *k_i_* is the permeability coefficient of material *i* and *L_i_* is the layer thickness of material *i*, as well as that the overall permeability coefficient for permeation in *PA*, *K_m,PA_*, is

(5) $$k_{m,PA}=\frac{\sum k_{i}A_{i}}{\sum A_{i}}$$

where *k_i_* is the permeability coefficient of material *i* and *L_i_* is the layer thickness of material *i*, it is easy to repeat the approach followed by Jin et al. [1], where the overall permeability of the specimen, *k_c_*, will be computed as

(6) $$k_{c}=\frac{d+H_{m}+d}{\frac{d}{k_{d}}+\frac{H_{m}}{k_{m,PA}}+\frac{d}{k_{d}}}$$

where *d* is the thickness of the deteriorated layer, *H_m_* is the thickness of the layer with *PA*, *k_d_* is the permeability coefficient of the deteriorated material and *k_m,PA_* is the equivalent permeability coefficient of the materials in *PA*, calculated from the following equation

(7) $$k_{m,PA}=\frac{k_{d}A_{d}+k_{0}A_{0}}{A_{d}+A_{0}}$$

where *k*_0_ is the permeability coefficient of the sound material, and *A_d_* and *A*_0_ are the areas of deteriorated and sound material, respectively, in the cross-section of the specimen, within *H_m_*.

Introducing Equation (7) in Equation (6) and making *A*_0_/(*A_d_* + *A*_0_) = *R_a_*, an equation equivalent to Equation (1) will be achieved.

However, considering the approach suggested in this Comment, a different *k_c_* is obtained. In this case

(8) $$k_{c}=\frac{k_{d}A_{d}+k_{m,SA}A_{0}}{A_{d}+A_{0}}$$

where *k_m,SA_* is the permeability coefficient of the materials in *SA*, calculated as

(9) $$k_{m,SA}=\frac{d+H_{m}+d}{\frac{d}{k_{d}}+\frac{H_{m}}{k_{0}}+\frac{d}{k_{d}}}.$$

Finally, introducing Equation (9) in Equation (8) and transforming variables in order to consider the same parameters as in Equation (1), results in

(10) $$R_{a}\left(1-2R_{H}\right)k_{d}{}^{2}+\left(k_{0}-k_{c}-k_{0}R_{a}+2k_{c}R_{H}+2k_{0}R_{a}R_{H}\right)k_{d}-2k_{0}k_{c}R_{H}=0.$$

**Figure 2:**
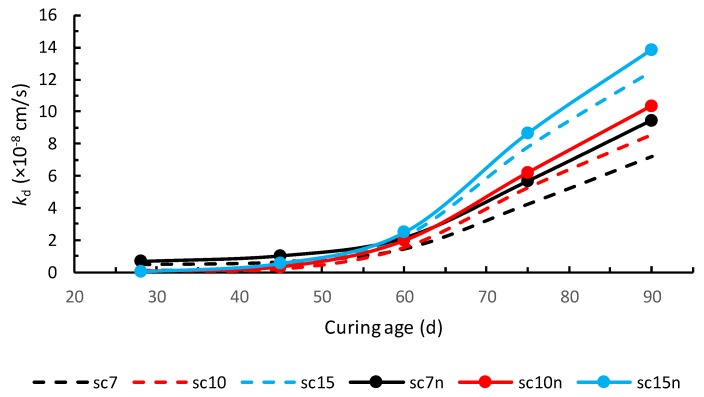
Comparison between the results presented in Reference [1] (sc7, sc10, sc15) with the results obtained from Equation (10) (sc7n, sc10n, sc15n).

Although Equation (10) is quadratic in *k_d_*, considering the available computation means, its numerical solution to find *k_d_* is advised, for the sake of reliability. In Figure 2, a comparison between the results presented in Reference [1] and those obtained with Equation (10) is shown.

A mean relative error of 35% was observed. Although it was not a considerable error, having presented the range of permeability coefficients in soil–cement, the scientific soundness requires the application of Equation (10) instead of Equation (1).

**Figure 3:**
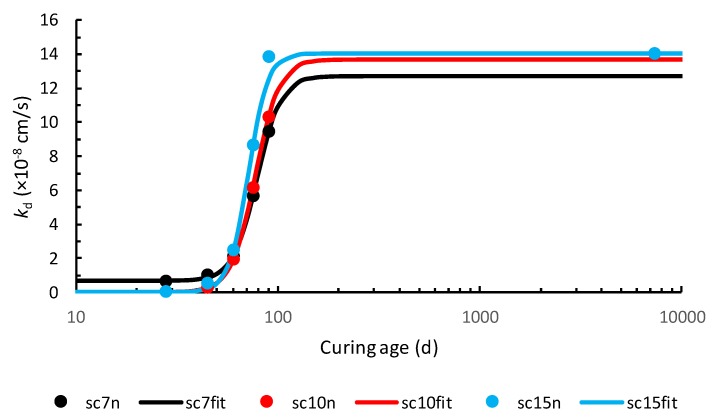
Fitting curves for the evolution of *k_d_* in time.

At last, an update of the function to model the evolution of the permeability coefficient of a deteriorated part of soil–cement is also recommended. Therefore, fitting Equation (12) of Reference [1], copied below, to the *k_d_* values computed from experimental results, through Equation (10), the parameters presented in Table 1 were obtained. The corresponding functions of permeability coefficient evolution in time are shown in Figure 3.

(11) $$k_{d}=\frac{k_{i}-k_{u}}{1+{\left(\frac{t}{t_{c}}\right)}^{p}}+k_{u}.$$

It shall be mentioned that the permeability coefficient considered for the soil–cement from Qingdao Port was 14.06 × 10^−8^ cm/s, corresponding to the mean value of 13.96 × 10^−8^ cm/s, 13.70 × 10^−8^ cm/s and 14.52 × 10^−8^ cm/s, instead of 14.41 × 10^−8^ cm/s. Furthermore, it is worth noticing that *k_u_* = 9.74 × 10^−8^ cm/s for soil–cement with a cement content of 15%, as found in Table 1 of Reference [1], cannot be correct, once there are two intermediate *k_d_* values (at *t* = 90 d and *t* = 7300 d) that are greater than it. 

## Figures and Tables

**Table 1:** Fitting parameters.

	*k_i_* (×10^−8^ cm/s)	*k_u_* (×10^−8^ cm/s)	*t_c_* (d)	*p*
sc7	0.68	12.72	78.57	7.29
sc10	0.04	13.67	76.97	7.26
sc15	0.04	14.06	71.21	9.07

## Notation

*A*_0_	Non-deteriorated area in the cross-section of the specimen
*A*_d_	Deteriorated area in the cross-section of the specimen
*d*	Deterioration depth
*H*	Total height of the specimen
*H_m_*	Height of the non-contaminated portion of the specimen
*k*_0_	Permeability coefficient of non-deteriorated soil–cement
*k_c_*	Overall permeability coefficient of soil–cement specimen
*k_d_*	Permeability coefficient of deteriorated soil–cement
*k_i_*	Lower asymptote of logistic function
*k_m,PA_*	Equivalent permeability coefficient for parallel association of materials
*k_m,SA_*	Equivalent permeability coefficient for serial association of materials
*k_u_*	Upper asymptote of logistic function
*p*	Shape parameter of logistic function
*PA*	Parallel association of materials
*R_a_*	$\frac{A_{d}}{A_{d}+A_{0}}$
*R_H_*	$\frac{d}{H}$
*SA*	Serial association of materials
*t*	Curing time
*t_c_*	Location parameter of logistic function

## References

[1] Jin Q., Cui X., Su J., Lu T., Wang J., Han R. (2019). Laboratory measurement and analysis of the deteriorated layer permeability coefficient of soil-cement deteriorated in a saline environment. Materials (Basel).

